# From Veld to Coast: Towards an Understanding of the Diverse Landscapes’ Uses by Past Foragers in Southern Africa

**DOI:** 10.1007/s41982-022-00124-w

**Published:** 2022-11-14

**Authors:** Aurore Val, Benjamin Collins

**Affiliations:** 1 Abteilung Für Ältere Urgeschichte Und Quartärökologie, Universität Tübingen, Tübingen, Germany; 2 Interdisciplinary Center for Archaeology and the Evolution of Human Behavior (ICArEHB), Universidade Do Algarve, Faro, Portugal; 3 Department of Anthropology, University of Manitoba, Winnipeg, Canada; 4 Department of Archaeology, University of Cape Town, Cape Town, South Africa

**Keywords:** Middle Pleistocene, Late Pleistocene, Holocene, Southern African archaeology, Palaeoenvironments, Human behaviours

## Abstract

In this brief contribution, we outline the aims of a collection entitled “From veld to coast: towards an understanding of the diverse landscapes’ uses by past foragers in southern Africa,” and we define its chronological, geographic and thematic framework.

Hominins, and especially members of our species, *Homo sapiens*, are characterized by adaptive behaviours that contribute to thriving in diverse environments (e.g. Kandel et al., 2016). To better characterize these behaviours in forager contexts, it is crucial to reconstruct past forager lifeways across the varied landscapes they inhabited. Southern Africa offers a unique opportunity to explore these questions because of two specificities: its remarkable ecological and climatic diversity, and the long time depth of foraging lifeways in the region.

From a modern, geopolitical point of view, southern Africa includes the countries located south of the Congo River Basin, namely: Angola, Botswana, Eswatini, Lesotho, Malawi, Mozambique, Namibia, South Africa, Zambia, and Zimbabwe (Fig. 1). The region is diverse geographically; it ranges from the low-lying coastal areas along the Indian and Atlantic oceanic shorelines to the high-altitude grasslands and savannahs of the Great Escarpment, with maximum elevations reached in the Maloti-Drakensberg Mountains from 2000 to 3482 m at Thabana-Ntlenyana, the highest peak in Africa south of the Kilimandjaro. Several large river systems irrigate the region, from the Zambezi River that forms the border between Zambia and Zimbabwe to the Senqu (Orange) River in South Africa (Fig. 2). The region comprises incredibly diverse vegetation biomes (Fig. 1), faunal cohorts, and mineral resources. The inland plateau is characterized by savannahs, grasslands and the semi-arid Succulent Karoo, Nama-Karoo, and Kalahari (Mucina & Rutherford, 2006). As one of several examples of southern Africa’s remarkable biodiversity, the Succulent Karoo represents the only arid biodiversity hotspot in the world (Myers et al., 2000). On the western fringe of the region, one of the oldest and driest deserts in the world, the Namib, stretches for more than 2000 km along the Atlantic coast, from the edge of the Karoo Biome in South Africa to the Moçâmedes Desert in southwest Angola. The Atlantic and the Indian oceans merge at the southwestern tip of the region; there, the confluence of different oceanic and atmospheric circulation systems, combined with the local geology, is associated with the presence of one of the five areas that form the global Mediterranean Biome occurring on the planet, the Fynbos. This biome is characterized by a remarkably high degree of plant and animal endemism. The Fynbos is home to several rock shelters with well-stratified, rich archaeological deposits associated with Late Pleistocene and Holocene human occupations. These sites have been instrumental in establishing the southern African Middle and Later Stone Age sequence (e.g., Lombard et al., 2012; Thackeray, 1992), as well as in demonstrating the unequivocal African origin and antiquity of technological and behavioural innovations previously attributed to later, European *Homo sapiens* (e.g., McBrearty & Brooks, 2000; Wadley, 2015; and references therein). To the east of the region, there is the narrow stretch of the subtropical Indian Ocean Coastal Belt Biome. The biome hosts remnant patches of Afromontane evergreen forest, which might have been more extensive in the past, prior human disruption of the endemic vegetation through farming (Mucina & Rutherford, 2006).

**Fig. 1:**
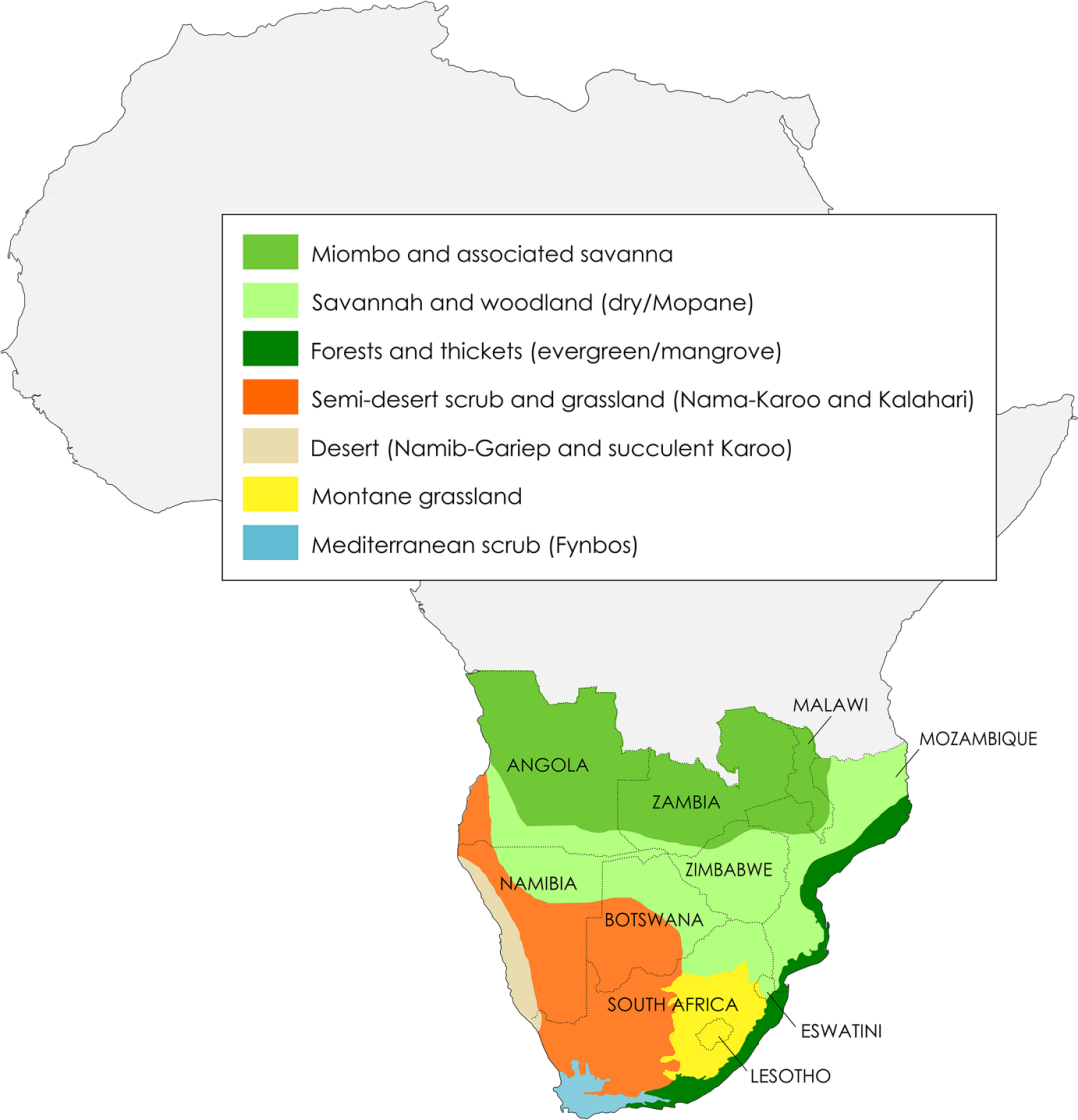
Modern political borders and vegetation biomes of southern Africa (redrawn and simplified from Biondi et al., 2015; Mucina & Rutherford, 2006; Stratford et al., 2021)

**Fig. 2:**
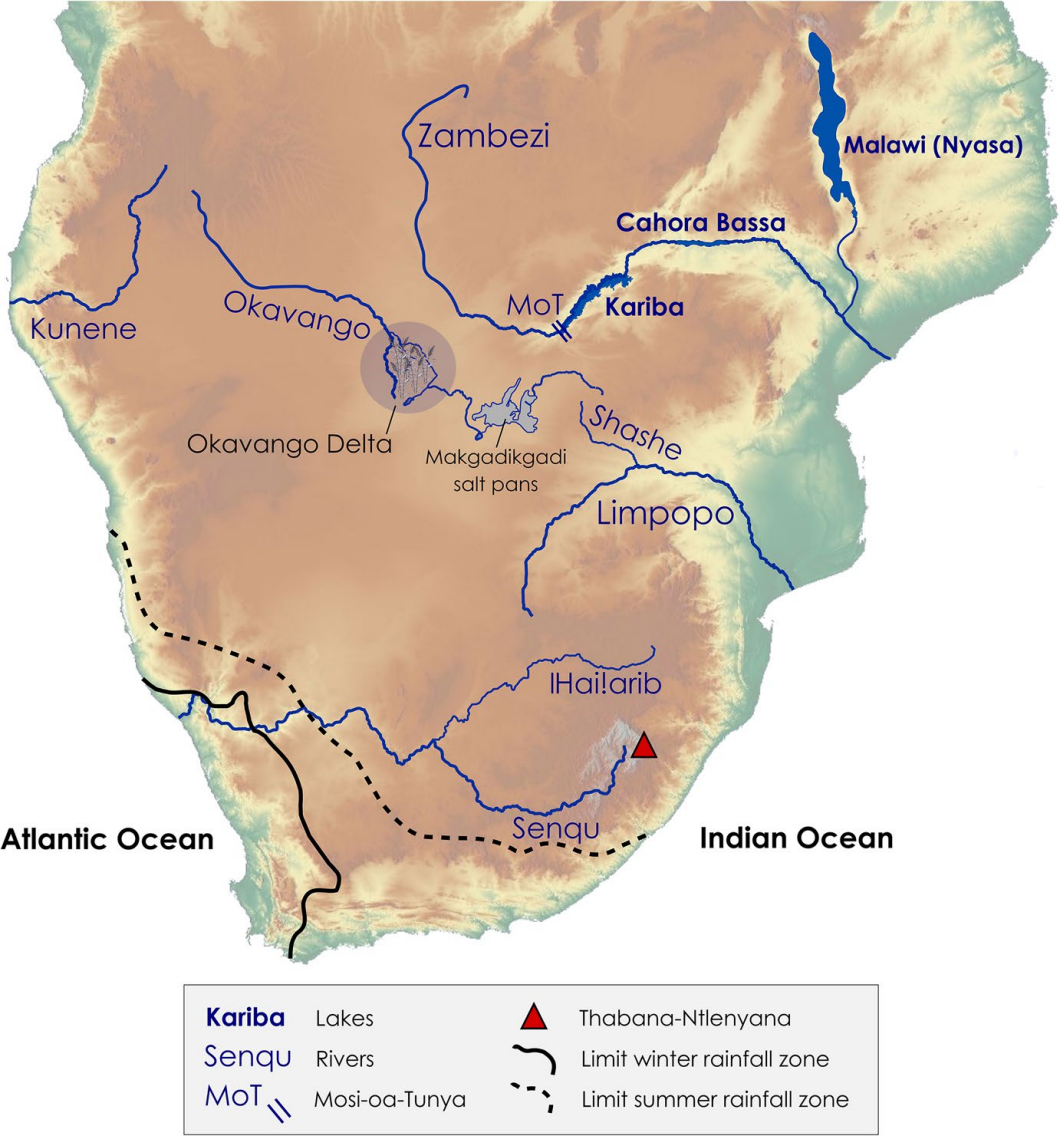
Large river systems, lakes, and relief and rainfall zones (after Chase & Meadows, 2007) of southern Africa

These different biomes have undergone major climatic variations during the Pleistocene and the Holocene, with, for instance, shifting of the extent of the winter rainfall zone (Chase & Meadows, 2007), and fluctuations of aridity in the interior through interglacial/glacial cycles (e.g., Lukich & Ecker, 2022). The palaeoclimatic record of southern Africa is patchy, sometimes contradictory, and in some cases markedly different from global climatic models (see for instance Chase, 2010; Urrego et al., 2015; Scott & Neumann, 2018), thus calling for a continuous effort to refine our knowledge of past climatic and environmental conditions at a local scale. Modern multidisciplinary archaeological research typically includes a strong palaeoenvironmental component and provides site-level resolution contributions to broader regional records (e.g., de la Peña et al., 2019; Lukich et al., 2020; Ames et al., 2020; Chazan et al., 2020; Marean et al., 2020 and associated contributions from that volume; Stratford et al. 2021 and papers therein; Mackay et al., 2022; von der Meden et al.,^ 2022^; Wroth et al., 2022). This approach allows for the development of higher resolution and more robust palaeoclimatic records at site and local levels and provides context for reconstructing and understanding past forager lifeways. In particular, palaeoenvironmental reconstructions provide a nuanced context to the exploration of the different behavioural strategies past peoples deployed to thrive in those environments, as well as setting the background for comparing archaeology both within and between different regions. Recent studies also highlight the role played by foragers in southern Africa to actively modify their ecosystem and carefully manage landscape resource (see notably Thompson et al., 2021; Davies et al., 2022). In this respect, the ongoing and complementary development of local and regional multiproxy palaeoenvironmental records is critical for refining models of past forager behaviour across southern Africa through time.

Although similarly patchy, the archaeological and fossil record of southern Africa testifies to the regular presence through time of humans and earlier hominins across the region (Fig. 3). Vast parts of southern Africa, particularly in the most arid areas of, for instance, Namibia and the South African interior, where erosion has exposed these artefacts on the surface, are littered with Earlier, Middle, and Later Stone Age artefacts (e.g., Mason, 1962; Sampson, 1968; Helgren & Brooks, 1983; Vogelsang, 1998; Nicoll, 2010; Marks, 2015; Hallinan, 2021; Phillips, 2022). This constitutes a clear indication that hominins have been thriving in these landscapes as far back as the beginning of the Pleistocene and, with regard to the Cradle of Humankind in South Africa, even earlier. This rich archaeological record lends itself to exploring regional-scale population dynamics through time, as well as interactions between trends in technology and social connectivity on the one hand and shifts in climate and environments on the other. In this respect, and building on a strong body of knowledge, this volume touches on several of the major focal points for forager research in southern Africa. Namely, it investigates how past peoples adapted to major climatic changes across glacial/interglacial cycles of the Late Pleistocene, understanding technological and social changes and diversity at local and regional scales, and exploring the transition from foraging to herding and farming lifestyles. The rich and diverse landscapes and archaeology provide unique opportunities to examine and discuss high-resolution archives from different sites across southern Africa. This approach is key for providing a more comprehensive and nuanced understanding of the major changes in forager behaviour through time.

**Fig. 3:**
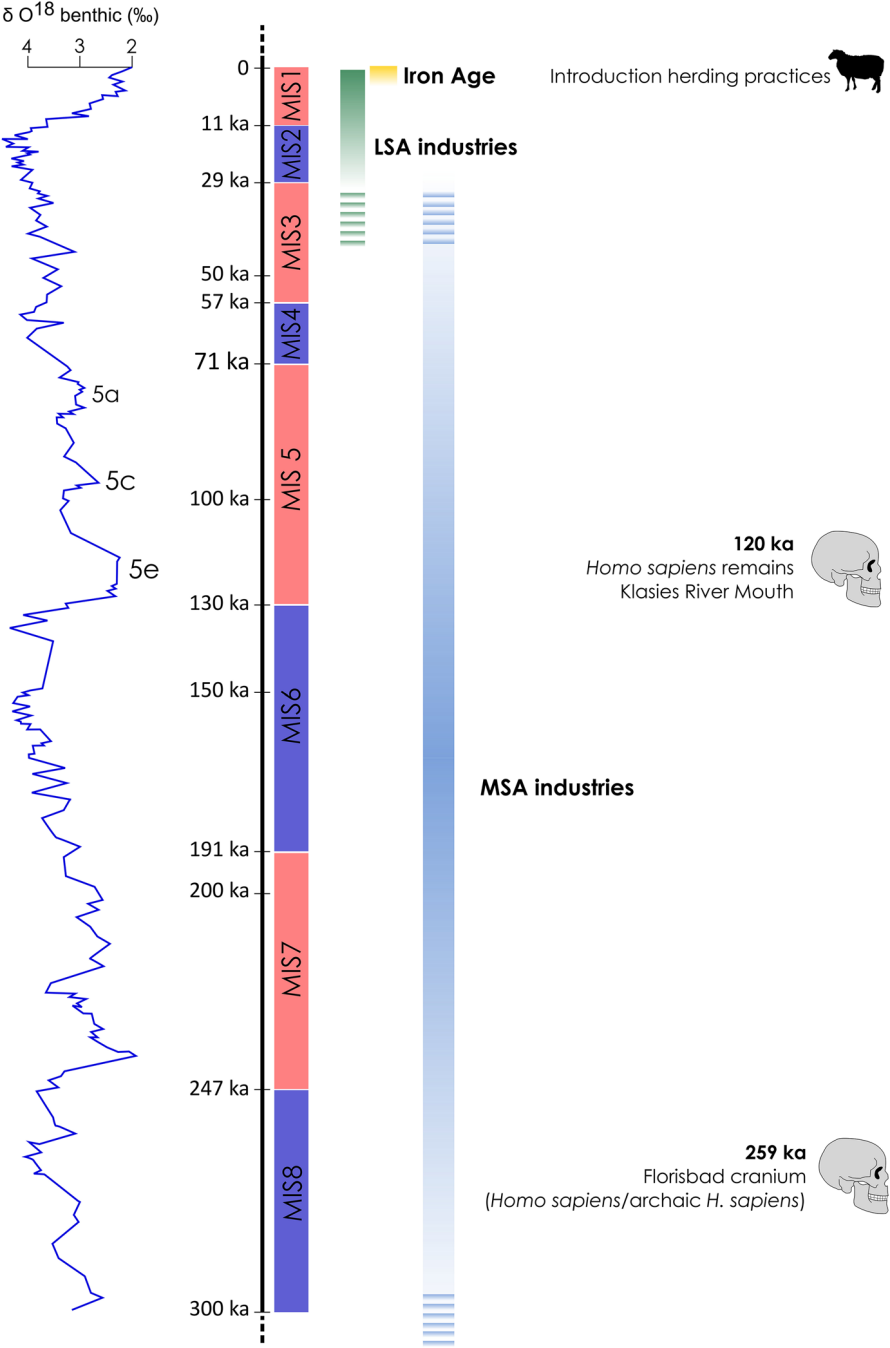
Chronology considered in the special issue with main cultural subdivisions and palaeoclimatic background. Left: oxygen isotopic composition of benthic foraminifera, simplified and redrawn from Urrego et al. (2015) (source: Bertrand et al., 2002) and Hayes et al. (2011) (source: Murray et al., 2000)

One issue with the rich southern African archaeological record is the bias towards specific regions in terms of research and contribution to the narrative of past foraging populations (e.g., Wilkins et al., 2021). These well-preserved and heavily researched regions, such as the humid southern coast of South Africa (e.g., Marean, 2014; Marean et al., 2007; Parkington, 2003) are crucial for the understanding of past foragers. Nevertheless, the focus on their role in shaping forager behaviour across the Pleistocene likely clouds our understanding of the diversity of past forager behaviours and adaptations, the range of landscapes in which past foragers thrived, and how they connected across these landscapes. Specifically lacking from this narrative are data from chronically understudied areas, including Eswatini, Zimbabwe, Angola, Malawi, Zambia, and Mozambique. This trend is changing however with recent research being reported from across understudied regions of South Africa (Dewar & Stewart, 2012; Stewart et al., 2012; Backwell et al., 2014; Porraz et al., 2015; Wadley et al., 2016; Collins et al., 2017; Backwell et al., 2018; de la Peña et al., 2019; Porraz & Val, 2019; Ames et al., 2020; Chazan et al., 2020; Val et al., 2021; Wadley et al., 2021; Wroth et al., 2022), as well as Eswatini (Bader et al., 2022), Angola (de Matos et al., 2022), Malawi (Thompson et al., 2012), and Mozambique (Gonçalves et al., 2016; Mercader et al., 2009). Several contributions to this volume focus on these understudied regions and emphasize the importance of going beyond sites, and thinking rather in terms of regions, and connectivity between regions, with regard to better understanding past foragers.

The climatic and environmental diversity of southern Africa translates into marked variations in plant, animal, and mineral resources, which would have been intimately interlaced with foragers’ subsistence practices and socio-economical organization. The exploration of still poorly documented regions of southern Africa is slowly helping to clarify the processes behind the emergence of distinct adaptive responses by foragers during the Middle to Late Pleistocene. This progress contrasts with the difficulty in highlighting any major difference in subsistence strategies between the Earlier and the Middle Stone Age (Smith et al., 2019).

With these questions in mind, we hosted a session entitled “From veld to coast: diverse landscape use by foragers in southern Africa from the Late Pleistocene to the Holocene,” initially intended to take place in April 2020 at the 85th Annual Meeting of the Society for American Archaeology in Austin, Texas. The COVID-19 pandemic postponed the meeting to 2021 and we held the session remotely. The format of the session, online and with the obligation to record talks several months in advance, prevented us from hosting as many participants as we had initially hoped and it proved somewhat frustrating, as discussions were limited to a chat feature during the session itself. Notwithstanding, the different contributions provided insights into ongoing research on Middle and Late Pleistocene human cultural adaptations across southern Africa (mostly South Africa). This prompted us to continue the discussion through a special journal issue on the topic. Our goal for this issue is to bring together results from a broad and diverse range of sites from across southern Africa to inform the behavioural diversity of Pleistocene and Holocene foragers in relationship to the various landscapes of the region. In this special issue, we welcome contributions providing new data on human interactions with all types of landscapes in the southern African region, as documented by their technological, behavioural, social, and symbolic expressions.

Another major theme that we invite the contributors to consider is the recurring use of ethnographic data in reconstructing past forager behaviours from Late Pleistocene to the more recent Holocene. This approach, and specifically the emphasis on ethnographic and historical accounts from Kalahari San groups, has, and remains, the focus of ongoing debate within the archaeological community (d’Errico et al., 2012; Pargeter et al., 2016 and replies; Mitchell, 2016). The historical contexts (Adhikari, 2011), variation in environments and resources, and substantial time depth augment for cautious use of these sources as heuristics for past behaviours (Wylie 1985), and especially so for sites and archaeological records that extend further back in time. Moreover, the contexts in which many of these sources were collected are problematic, with no clear indication of informed consent and very clear power dynamics that disadvantaged and exploited Indigenous peoples to extract their knowledge. In this respect, we encourage the contributors to start thinking outside of the ethnographic box and explore alternative approaches to developing heuristics for interpreting and understanding past forager behaviours in southern Africa.

This special issue will encompass a range of contributions as spatial-temporally and thematically broad as possible, in order to contribute to the growing body of archaeological research work in southern Africa. Specifically, contributions in this issue will actively contribute towards the following:

- Building local chrono-cultural sequences (site-scale, biome-scale)
- Refining local palaeoenvironmental and chronological data to provide better contextual data to the archaeological evidence
- Exploring and documenting areas historically understudied
- Exploring factors behind technological variability and change through time
- Evaluating the efficacy and use of the ethnographic data for understanding past foragers from diverse landscapes
- Questioning “transitions” (MSA/LSA, Pleistocene/Holocene, LSA technology/herding, and farming practices)
- Questioning the nature of human interactions within the region, through time (connectedness versus isolation, diffusion, innovation, population dynamics, existence of geographical barriers, corridors, etc.)

It is important to note that these themes are not silos, they are strongly interconnected, and together they can provide powerful insight into how past foragers thrived across southern Africa’s diverse landscapes through time. In this respect, we are excited for the forthcoming contributions, as well as the final concluding paper, which will be co-authored by all of the contributors in this volume. This approach, and credit to the Journal of Paleolithic Archaeology for this innovative format, will facilitate a lively discussion of the themes mentioned above. In doing so, this will hopefully move the conversation along from dated topics, such as behavioural modernity (Shea, 2011; Ames et al., 2013; Porr and Matthews 2019), to a more nuanced and coherent understanding of diverse forager behaviours across space and through time.

## Data Availability

Not applicable.
